# Pain Management for Nerve Injury following Dental Implant Surgery at Tokyo Dental College Hospital

**DOI:** 10.1155/2012/209474

**Published:** 2012-07-30

**Authors:** Ken-ichi Fukuda, Tatsuya Ichinohe, Yuzuru Kaneko

**Affiliations:** ^1^Division of Dental Anesthesiology, Department of Oral Health and Clinical Science, Tokyo Dental College, (Suidoubashi Hospital, Dental Anesthesia/Orofacial Pain Center), 2-9-18, Misaki-cho, Chiyoda-ku, Tokyo 101-0061, Japan; ^2^Department of Dental Anesthesiology, Tokyo Dental College, 2-9-18, Misaki-cho, Chiyoda-ku, Tokyo 101-0061, Japan

## Abstract

By allowing reconstruction of compromised occlusion, dental implants contribute to an improvement in quality of life (QOL) and diet. Injury to a nerve during such treatment, however, can result in a sudden decline in QOL. And once a nerve has been injured, the chances of a full recovery are slim unless the damage is only slight. If such damage causes neuropathic pain severe enough to prevent sleep, the patient's QOL will deteriorate dramatically. While damage to skin tissue or bone invariably heals over time, damage to nerves does not, indicating the need to avoid such injury while performing implant insertion, for example. This means not relying solely on X-ray images, which can be rather unclear, but also using computed tomography to allow preoperative planning and intraoperative execution to be performed as accurately as possible. Moreover, if sensory damage does occur it is essential to avoid breaking the bond of trust between dentist and patient by giving false assurances of recovery. In such cases, appropriate measures must be taken promptly. This paper describes pain management for nerve injury following dental implant surgery at the Orofacial Pain Center of Tokyo Dental College Suidoubashi Hospital.

## 1. Introduction

Implants are used to reconstruct compromised occlusion, resulting in an improvement in QOL and diet. Such dental implant surgery itself, however, may damage the nerves, resulting in a sudden decrease in QOL. Moreover, once such damage has occurred, a complete recovery is rare unless the extent of the injury is only minor. The interaction between the peripheral sensory nerves and the central nervous system (CNS) is extremely complicated, unlike that with bone or mucous membrane. The way information is relayed between the nerves and the CNS is complex and can easily be disrupted. A minor injury such as a bite may only result in numbness. More severe injury, however, may result in dysesthesia or neuropathic pain, causing sustained discomfort, especially at night, and severely affecting QOL.

In this paper, we will explain what happens following a nerve injury and the mechanism of neuropathic pain. Furthermore, we will describe how pain is managed following such injury incurred during dental implant surgery at our pain clinic.

## 2. Process following Nerve Injury 

If an implant fixture causes damage to the inferior alveolar nerve at the time of implant placement, what happens to the damaged tissues? How is the damaged nerve restored? And what changes occur in the damaged nerve tissue?

**Figure 1:**
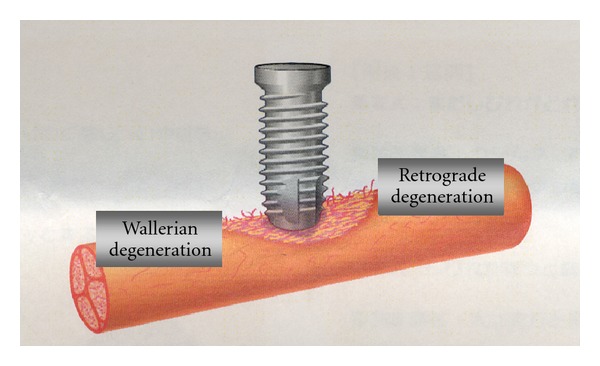
Degeneration following nerve injury by implant fixture [18].

When the inferior alveolar nerve fibers are damaged by implant fixtures, retrograde degeneration toward the CNS and Wallerian degeneration toward the periphery start from around the site of the injury within just a few minutes (Figure 1). Even if the nerve fibers become disconnected, they will be rapidly reconnected when the implant fixtures are removed. A linear Schwann cell array is observed between the disconnected nerve fibers, which significantly increases nerve growth factor and its family groups. In addition, genes encoding receptor mRNA show a marked increase and nerve regeneration is activated. These defense mechanisms and repair reactions can be completed within 2 to 3 weeks. That is to say, the nerves are reconnected quite quickly unless neuropathy is severe or severance has occurred. Although connections may recover histologically, however, function does not.

**Figure 2:**
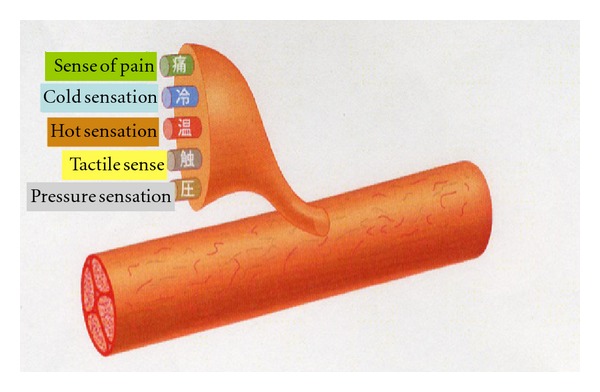
Various sensations in sensory nerve [18].

**Figure 3:**
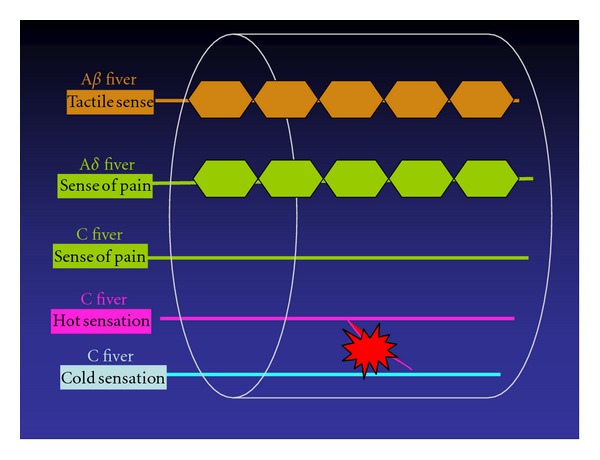
Ephapse between cold and warm sensation.

Reconnection does not mean wound healing. On the contrary, reconnection under such conditions may cause distress to the patient. The inferior alveolar nerve is complex, and is responsible for algesthesia and sensitivity to touch, heat, cold, and pressure, although the mechanism in each is different. One analogy would be the underground pipe network in a big city, which carries gas, electricity, telephone, water, and sewage, but each separately and by a different route (Figure 2). If nerve networks are damaged, the insularity of the structure is compromised, and when regeneration takes place, adjacent nerve fibers may be accidentally connected. This means that impulses from the peripheral nerves may be transmitted to the wrong destination, and from there on to the CNS. If we take the previously mentioned analogy, it is as if underground water and gas pipes broken due to an earthquake have been cross-connected. Such inappropriate connection has been termed “ephapse” [1], and has been proved in a laboratory animal model [2]. That is to say, nerve function never completely recovers, even if nerve regeneration is histologically completed through active nerve regeneration after the injury. For example, let us take the case of nerve fibers intended for transmission of cold sensation being accidentally connected with those intended to transmit heat sensation (Figure 3). This would compromise a patient's ability to notice and report change in temperature. There are countless nerve fibers, so injury to any specific nerve may result in connection to any number of other nerves depending on the severity of the injury involved. A large number of inappropriate nerve fiber connections often results in neuroma formation. Therefore, the presence of a neuroma indicates possible nerve damage and ephapse. Neuromas often exhibit spontaneous discharge [3]. This phenomenon is termed “dysesthesia” in the patient, who will typically complain of sensations of numbness, tingling, or something cold on a limb, for example.

## 3. Mechanism of Neuropathic Pain

**Figure 4:**
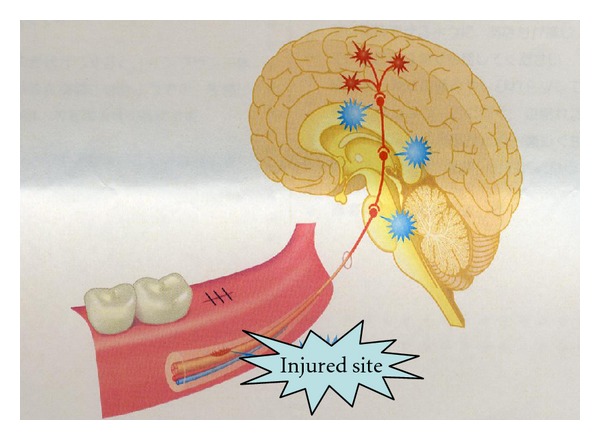
Influence of nerve injury on all neurons [18].

**Figure 5:**
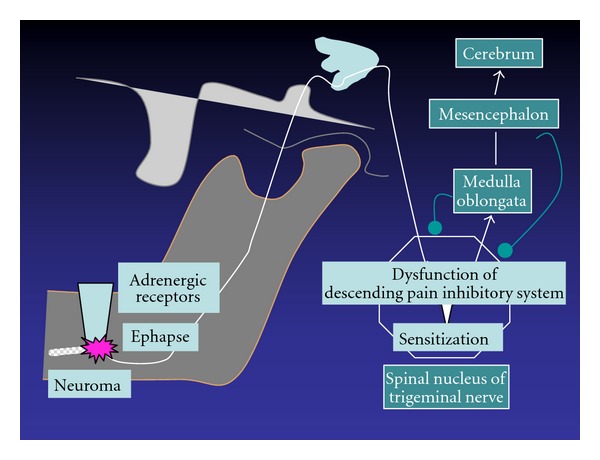
Pathogenic mechanism of neuropathic pain [19].

Regressive change in axons and the myelin sheath and nerve regeneration influence all neurons, not only histologically, but also molecularly and electrophysiologically. In other words, the healing of a damaged nerve is not limited to the site of the injury alone, but involves the entire system from the periphery to the center, unlike with mucous membrane or bone. Thus, the healing of a nerve injury is more complicated. Sensory nerves are variously modified until sensory information from the periphery nerves reaches the cerebrum (Figure 4). Pain in the region of the inferior alveolar nerve is transmitted from the peripheral nerves to the third branch of the trigeminal and mandibular nerves, and from there to the spinal nucleus of the trigeminal nerve and several neurons, finally reaching the somatosensory cortex, where it is then recognized. Various neural networks are involved in this pathway, including the descending pain inhibitory system, the excitatory system, and the sympathetic nervous system, involving emotion, which modifies the sensory nerves. These systems are complicated, and therefore information transmitted is severely distorted if the transduction mechanism is damaged. For example, when the descending pain inhibitory system, which acts as a brake, does not work, or when the excitatory system, which acts as an accelerator, runs out of control, information which induces severe pain is transmitted. This is known as neuropathic pain, defined as “Pain initiated or caused by a primary lesion or dysfunction in the nervous system” by the International Association for the Study of Pain. The onset mechanism of neuropathic pain is classified into three parts: peripheral neuropathic pain, sympathetically maintained neuropathic pain, and central neuropathic pain (Figure 5).

Ephapse formation may be involved in the peripheral mechanism, as mentioned above. When nerve fibers that transmit algesthesia and those that transmit the tactile sense are connected, the patient will experience pain on touch, some even experiencing very severe pain when swallowing food. As mentioned above, it is suspected that spontaneous discharge from neuromas and the development of dysesthesia are deeply involved in the onset of neuropathic pain. Ephapse formation activates retrograde conduction by axon reflex, which in turn results in secretion of substance P and calcitonin gene related peptide from nerve terminals. Moreover, axon reflex induces an increase in the sprouting of nociceptors in the nerve terminals and subsequent sensitivity to pain. Such hypersensitivity remains, even after the damaged nerves are histologically repaired. This type of pain exacerbation in the peripheral nervous system is called peripheral sensitization.

Next let us move on to the sympathetically maintained mechanism. Regenerated nerve endings are more sensitive to catecholamine receptors after nerve injury [4]. In addition, generation of sympathetic nerve fibers is observed in dorsal root ganglia [5]. Information on pain accelerates the neural reflex from the spinal nucleus of the trigeminal nerve to the sympathetic efferent pathway. Sympathicotonia is transmitted by reflex action to the peripheral nervous system, inducing peripheral vascular compromise and an increase in pain sensation, which in turn exacerbates the sympathicotonia, forming a vicious cycle.

Now, let us look at the onset mechanism of central neuropathic pain. In the spinal nucleus of the trigeminal nerve, structural change in cell membranes by continuous stimulation from the peripheral nerves, such as the windup phenomenon and excitatory amino acid receptor, produces incorrect information, causing severe pain, which is known as central sensitization [6]. The descending pain inhibition system usually functions with it in a coordinated manner, but it falls into ataxia quite frequently following nerve injury. Neuropathic pain is established by the aforementioned mechanism [7]. In general, neuropathic pain is intricately involved in various such mechanisms. The size of the injury, inflammation due to infection, and genetic conditions are all possible factors in the development of pain or dysesthesia after nerve injury, but the precise role of each remains to be determined.

## 4. Category of Nerve Injury

While dysesthesia is characterized by spontaneous or evoked discomfort, neural pain, known as allodynia, is caused by slight contact or compression or stimulation to light to usually cause pain. Neuropathy is classified into 3 main types: axonotmesis, neurotmesis, and neuropraxia [8]. Neurotmesis indicates rupture of the nerve trunk where both the axon and axon continuity is completely severed, while axonotmesis indicates the rupture of axons where the surrounding connective tissue of the nerve has been maintained. Neuropraxia is a transient blockage of nerve conduction without axon alteration. Of course, the prognosis varies depending on severity. If nerve compression and ischemia due to swelling, bleeding, or vascular compromise leads to neuropraxia, the patient will be able to recover in a few months; if the result is neurotmesis or axonotmesis, however, the patient is unlikely to ever completely recover.

## 5. Preparation and Prevention

**Figure 6:**
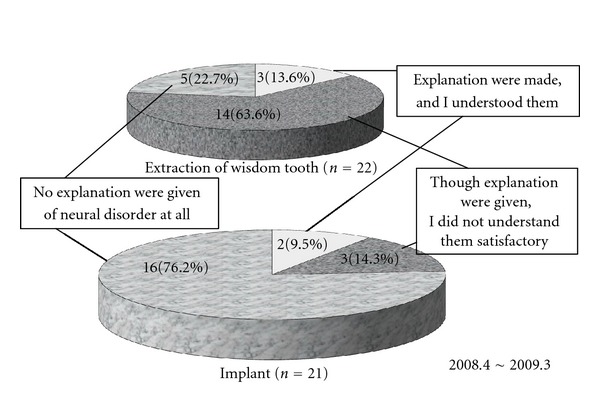
Questionnaire on preoperative informed consent [18].

Whether performing dental implant surgery or wisdom tooth extraction, dentists have to explain to the patient the possibility of adverse events and reactions before commencing in order to obtain informed consent.  Figure 6 shows the results of a survey of 22 patients undergoing wisdom tooth extraction and 21 patients who developed inferior alveolar neuropathy after dental implant surgery at the Department of Dental Anesthesia, Tokyo Dental College Suidobashi Hospital between April 2008 and March 2009. Of these patients, only 5 patients undergoing dental implant surgery (23.8%) received a preoperative explanation about the possible onset of neuropathy. Of these 5 patients, 3 patients did not understand the explanation well. This percentage was even lower in patients undergoing wisdom tooth extraction. This indicates the need to explain the potential problem of neuropathy and how it might be avoided in a way that can be understood by the layman. 

These days, preoperative CT imaging is essential for patients undergoing implant placement surgery. Of the 21 patients who developed inferior alveolar neuropathy after dental implant surgery in the present survey, only 5 patients underwent preoperative CT imaging (23.8%). This indicates the need for CT imaging to be performed as part of the preoperative workup.

The causes of nerve injury by implant fixtures include direct injury during drilling, compression by the implant fixture itself, hematoma, edema, aberrant mandibular nerve canal, and reactive bone augmentation. The upper layer of bone surrounding the mandibular canal is thin and missing in some areas, making it prone to break if peri-implantitis should occur. Moreover, submerged implant fixtures can also cause damage. Therefore, the degree of clearance from the top of the mandibular canal should be taken into consideration when planning implant placement. In general, the drill used in implant placement is approximately 1 mm longer than the implant fixture to be used, and the height of mandibular alveolar crest is different between the lingual and buccal sides. Thus, the depth of the tooth socket on the X-ray image and the actual implant placement depth may differ. This indicates the need to check both before commencing surgical procedures. It is recommended that a clearance of at least 3 mm be left from the top of the mandibular canal [9]. Clearance should be set according to the experience of the dentist involved.

## 6. What If the Nerve Is Damaged? 

**Figure 7:**
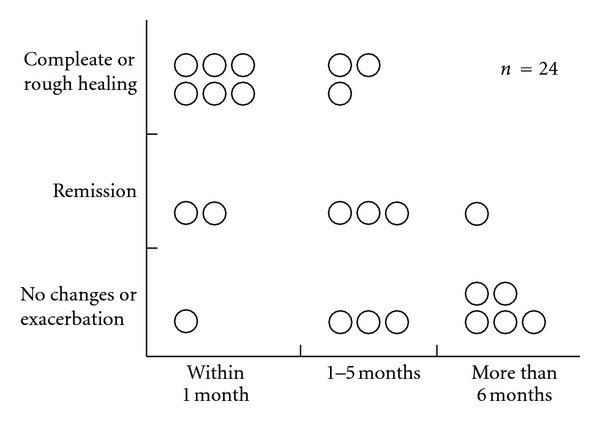
Prognosis depending on time of initiation of treatment [19].

Wallerian degeneration is minimized within 1 to 2 weeks after damage occurs, and it is important to promote nerve regeneration in an appropriate direction in order to obtain a good prognosis. Therefore, if the nerve is damaged, medical evaluation and treatment should be performed as soon as possible. Specialist attention should be sought within 1 to 2 weeks after damage occurring and treatment commenced.  Figure 7 shows the difference in prognosis by treatment start date. The number of patients who achieved complete remission, meaning an almost complete recovery, increased the earlier treatment started, whereas the majority of those in whom treatment was commenced at 6 months or more after nerve injury occurred showed either no change or exacerbation. If the nerve is damaged, evaluation and treatment should be started within a month to obtain a good prognosis. 

## 7. Assessments for Neuropathy

**Figure 8:**
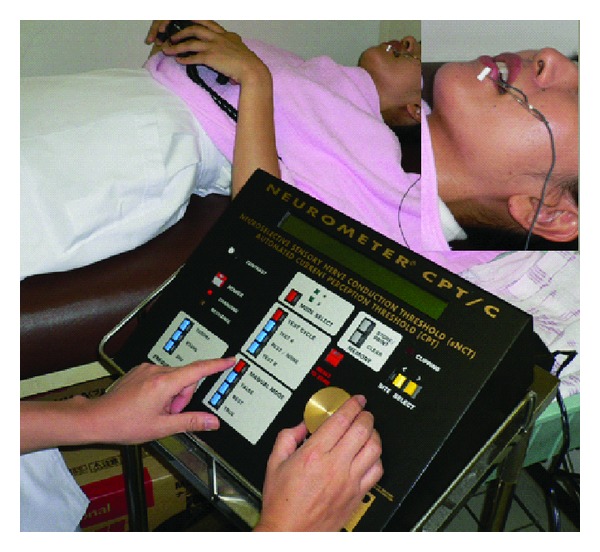
Current perception threshold.

**Figure 9:**
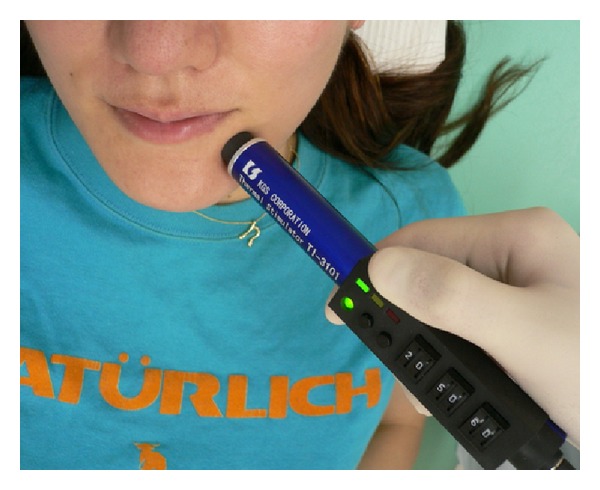
Nerve testing for hot or cold sensation.

There are various tests available which allow the patient's sense of touch, temperature, cold, and pressure to be assessed or the presence of algesthesia to be determined. Therefore, location and severity can be determined objectively. In addition, X-ray examination and CT imaging to precisely locate the mandibular canal and the presence of any foreign bodies, and MRI to visualize nerves and tissues which cannot be observed by X-ray are also available. A tactile sense test can include a touch detection test (Semmes-Weinstein Aesthesiometer test), static two-point discrimination, and assessment of recognition using an electric detective threshold. In addition, a Neurometer (Neurotron Incorporated, U.S.) may be used to assess current perception thresholds at frequencies of 2000, 250, and 5 Hz, which selectively stimulate A**β**, A**δ**, and c fibers, respectively, and determine the presence or absence of fiber-based nerve injuries (Figure 8).  Figure 9 shows hot or cold sensation testing (KGS Incorporated, Japan). This device can be used to examine function in temperature receptors. With this device, temperatures can be adjusted to determine at what point a person perceives hot or cold as pain. 

**Figure 10:**
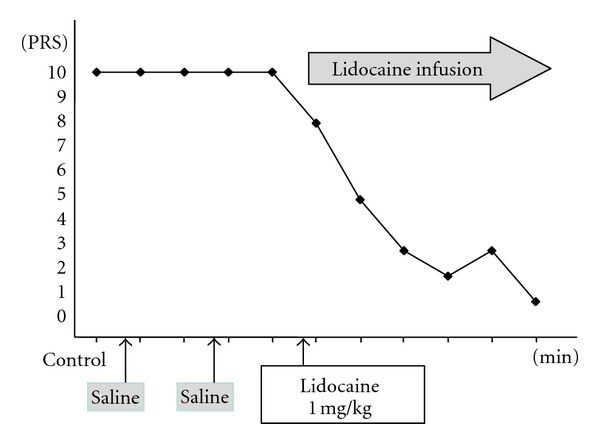
Drug challenge test [10].

The functioning of pain receptors is examined by measuring the pain threshold using a device which imparts a load using a spring and a weight. As described above, if the conditions are complicated and various mechanisms are involved, neuropathic pain may become intractable. To determine whether chronic neuropathic pain is caused by a peripheral, central, or sympathetically maintained mechanism, or all of them, a drug challenge test is used [10]. In this type of test, exploratory intravenous infusion of drugs such as lidocaine, ketamine, phentolamine, and morphine, whose analgesic mechanism has already been elucidated, is used to investigate onset and change in severe pain which is refractory to treatment, including neuropathic pain. For example, a single dose of 1 mg/kg lidocaine is intravenously administered, followed by continuous intravenous administration of 1 mg/kg lidocaine over 30 minutes. The pain relief response is then observed using VAS or NRS at 1, 3, 5, 10, 15, 20, 25, and 30 minutes after start of infusion (Figure 10). If the pain relief response is clear, the onset mechanism of pain may represent the firing of injured nerve fibers. Intravenous infusion of lidocaine, mexiletine, or oral lidocaine, or a lidocaine cream is also used. A complete pain relief response with administration of a single dose of 5 mg ketamine may indicate that it was antagonized by glutamic acid and NMDA receptors, suggesting central neuropathic pain. Continuous intravenous infusion of ketamine or oral ketamine is used for pain relief. If phentolamine works for pain relief, the pain may be sympathetically maintained, in which case Stellate ganglion block is performed. Thus, it is important to select the appropriate treatment method by understanding the type of neuropathic pain involved.

**Figure 11:**
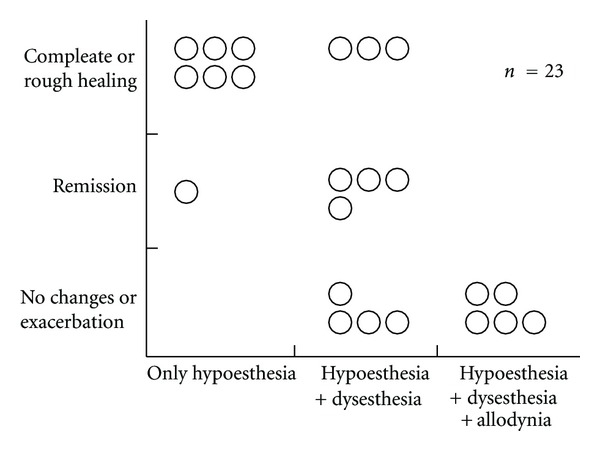
Prognosis and subjective symptoms [19].

It is also important to evaluate subjective symptoms for estimation of prognosis. If the subjective symptom is hypoesthesia alone, the prognosis will be good. If dysesthesia and allodynia also develop, the prognosis will be poor and neuropathic pain is likely to develop (Figure 11). Thus, if dysesthesia and/or allodynia are observed, treatment should be performed promptly.

According to some recent reports [11], if a patient experiences no signs of an improvement or develops dysesthesia at even 1 week after nerve injury, the outlook for a spontaneous recovery is poor. 

## 8. Treatments for Neuropathy

**Figure 12:**
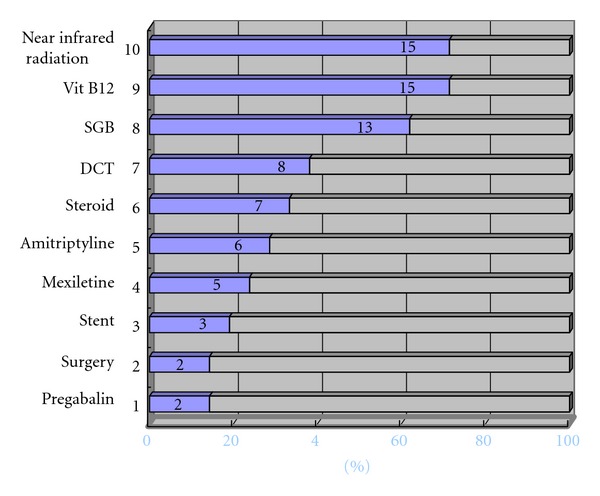
Methods of treatments after nerve injury used at Tokyo Dental College Suidoubashi Hospital (2008.4-2009.3) SGB: stellate ganglion block; DCT: drug-challenge test [18].

**Figure 13:**
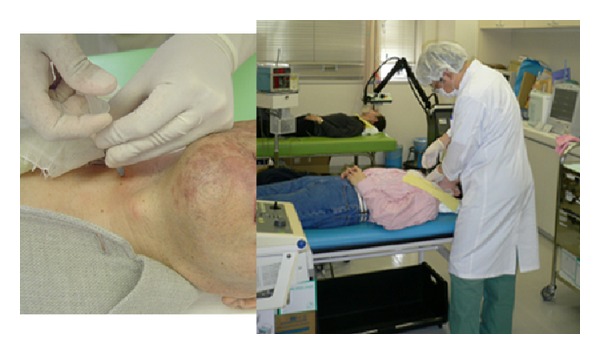
Stellate ganglion block.

**Figure 14:**
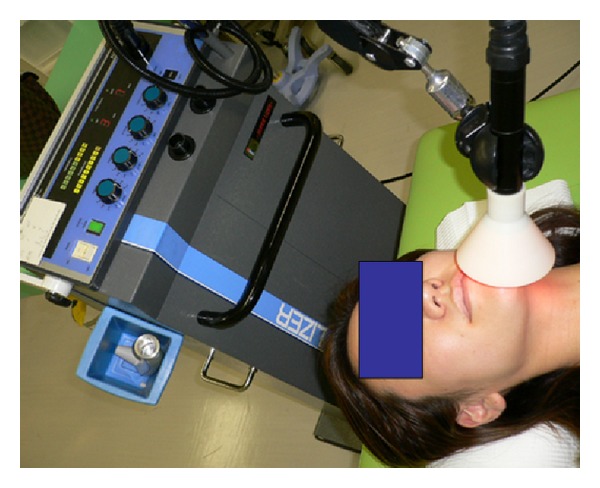
Near-infrared therapy.

Treatment for neuropathy includes near-infrared therapy, stellate ganglion block, medication (oral, infusional, or topical), traditional Chinese medicine, acupuncture, and surgery.  Figure 12 shows which treatment methods were used until December 2010 in 21 patients visiting the Outpatient Department of Anesthesiology, Tokyo Dental College Suidobashi Hospital between April 2008 and March 2009 with inferior alveolar neuropathy secondary to dental implant surgery. Treatment varies depending on the length of time between onset of neuropathy and initial examination. Patients who visit the hospital at an early stage receive intense treatment to promote neurologic recovery. Patients with chronic neuropathy receive treatment to relieve subjective symptoms. A radical, infusional procedure, stellate ganglion block (SGB) is applied in both types of patient (Figure 13). It blocks the sympathetic nervous system to increase blood flow and prevent edema in the nerve distribution. In rabbit studies [12, 13], common carotid arterial, tongue mucosal, mandibular bone marrow, and masseter muscle blood flow all showed an increase following SGB, as did tissue oxygen tension on the block side. This increase in blood flow promotes nerve fiber regeneration, that is, promotion of neurologic recovery. This procedure is usually performed at least 1 to 2 months after nerve injury. During this period, the injured nerve is actively repaired. In a rat study [14], sympathetic nerve block at an early stage accelerated the neurophysiological recovery and regeneration of severed infraorbital nerves. Some patients with neuropathic pain caused by nerve injury suffer from severe sympathetically maintained pain. These patients undergo SGB to block the sympathetic nervous system for relief of pain and dysesthesia. Medication includes Vitamin B12 to promote regeneration of nerve terminals, ATP to increase blood flow by vasodilation, and steroids to reduce neuritis and edema. Near-infrared therapy is also effective in increasing regional blood flow (Figure 14). Stellate ganglion block also inhibits secondary central sensitization, which prevents neuropathic pain.

**Figure 15:**
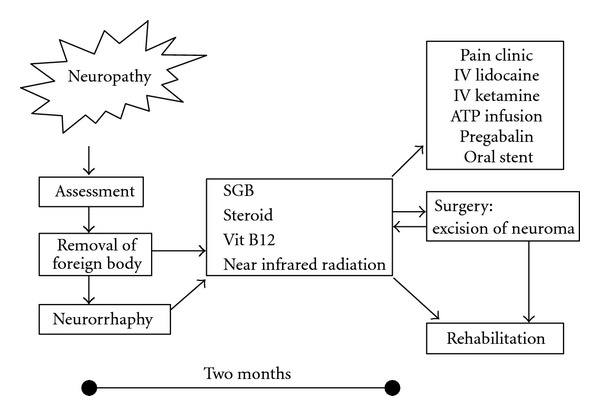
Therapies for neuropathy [18].

Patients with nerve injury occurring 1 to 2 months prior to visiting our hospital are given intensive treatment with a combination of these therapies (Figure 15). If neuropathic pain or allodynia develops following nerve injury, a tricyclic antidepressant to stimulate the descending pain inhibitory system, an antiepileptic agent to inhibit abnormal neuronal excitation, IV Infusion with ketamine, lidocaine, or ATP [15], and stent capsaicin are performed to control pain. If symptoms do not improve or pain persists for several months after injury, surgery is performed. Meyer et al. [16] reported that the success rate decreased when surgery was performed 6 months or more after the injury. Robinson et al. [17] also reported that three months was the critical point for postinjury intervention, indicating the need to act quickly in such cases. At Tokyo Dental College Suidobashi Hospital, implant fixtures are closely examined in patients who develop nerve injury relatively soon after implant placement. If the fixtures are identified as the cause of the injury, they are removed. However, it is not necessary to remove the implant fixtures in all cases. We should avoid exacerbating the injury as a result of the removal procedure. If neurotmesis, that is to say, axonotmesis, develops, neurorrhaphy is performed first. Stellate ganglion block, administration of corticosteroids and vitamin B12, near-infrared therapy, and/or warm compresses at home are all prescribed in patients with nerve injury of 1 to 2 months duration. If the pain continues for more than 3 months, pain treatment is performed. If the symptoms are exacerbated rather than ameliorated, surgical removal of the neuroma is discussed with the patient.

Neuropathic symptoms caused by sensory nerve injury are generally intractable. Moreover, in some cases, the nerve injury may be asymptomatic; in others, the disease may be considerably prolonged; and a common problem is the failure of family and friends to understand the patient's distress. In such cases, it is not uncommon for psychiatric disorders such as anxiety neurosis and depression to develop. Not only the medical/surgical treatment, but also the psychological care of such patients is crucial. Therefore, it is sometimes necessary to consult with a psychiatrist or psychotherapist as to whether psychosomatic medicine should also be applied.
